# Factorial Analysis and Thermal Kinetics of Chemical Recycling of Poly(ethylene terephthalate) Aided by Neoteric Imidazolium-Based Ionic Liquids

**DOI:** 10.3390/polym16172451

**Published:** 2024-08-29

**Authors:** Oscar Gil-Castell, Ramón Jiménez-Robles, Alejandro Gálvez-Subiela, Gorka Marco-Velasco, M. Pilar Cumplido, Laia Martín-Pérez, Amparo Cháfer, Jose D. Badia

**Affiliations:** 1 Research Group in Materials Technology and Sustainability (MATS), Department of Chemical Engineering, School of Engineering, Universitat de València, Av. Universitat s/n, 46100 Burjassot, Valencia, Spain; oscar.gil@uv.es (O.G.-C.);; 2 Plastic Technology Centre (AIMPLAS), Gustave Eiffel 4, 46980 Paterna, Valencia, Spain; pcumplido@aimplas.es

**Keywords:** plastic waste, valorization, chemical recycling, glycolysis, poly(ethylene terephthalate) (PET), neoteric solvents, ionic liquid

## Abstract

Poly(ethylene terephthalate) (PET) waste accumulation poses significant environmental challenges due to its persistent nature and current management limitations. This study explores the effectiveness of imidazolium-based neoteric solvents [Emim][OAc] and [Bmim][OAc] as catalytic co-solvents in the glycolysis of PET with ethylene glycol (EG). Reaction thermal kinetics showed that both ionic liquids (ILs) significantly enhanced the depolymerization rate of PET compared to traditional methods. The use of [Emim][OAc] offered a lower activation energy of 88.69 kJ·mol^−1^, thus making the process more energy-efficient. The contribution of key process parameters, including temperature (T), plastic-to-ionic liquid (P/IL) mass ratio, and plastic-to-solvent (P/S) mass ratio, were evaluated by means of a factorial analysis and optimized to achieve the maximum PET conversion for both neoteric solvents. The relevance sequence for both ionic liquids involved the linear factors T and P/S, followed by the interaction factors T×P/S and T×P/IL, with P/IL being the less significant parameter. The optimal conditions, with a predicted conversion of 100%, involved a temperature of 190 °C, with a P/IL of 1:1 and a P/S of 1:2.5, regardless of the IL used as the catalytic co-solvent.

## 1. Introduction

The global annual production of plastics recently exceeded 400 million tons, with the packaging sector representing a significant portion of this demand. In Europe, over 37 million tons of plastics are currently produced per year, more than half of which become post-consumer waste. Despite efforts to manage post-consumption plastics through mechanical recycling (27%), or with energy recovery strategies (50%), a substantial percentage still ends up in landfills (23%) [1].

Poly(ethylene terephthalate) (PET) is the most relevant linear aromatic polyester and ranks as the fourth most utilized polymer, with a prominent role in the packaging sector due to its durability, versatility, and lightness [2]. However, the widespread use of PET for single-use products generates significant environmental and economic concerns [3], highlighting the necessity for sustainable PET waste management, in line with circular economy principles [4,5,6].

Recent progress in PET valorization includes advanced mechanical recycling strategies [7,8,9,10], enzymatic depolymerization [11,12], or radiation-assisted chemical recycling [13,14]. Notwithstanding these innovations, there are still technology readiness limitations or process drawbacks that underscore the need for alternative technological solutions to enhance the circularity of plastic waste [15]. Mechanical recycling struggles with contamination, quality control, and mechanical and thermal degradation during reprocessing [9]. Enzymatic depolymerization encounters scale-up and cost issues, along with higher reaction times. Radiation-assisted chemical recycling requires specialized and complex equipment, safety protocols, and can be energy-intensive. Thus, combining valorization technologies [16] and/or developing complementary recycling technologies is essential to meet the circular economy goals, the United Nations 2030 Agenda for Sustainable Development [17], and the United Nations Sustainable Development Goals (SDGs) [18].

Chemical recycling breaks down the polymer chain through depolymerization, producing raw monomers or oligomers that can be used for new polymer synthesis and generating other valuable by-products [19,20,21]. Several prominent chemical recycling methods for PET include glycolysis, hydrolysis, methanolysis, aminolysis, and hydrogenation, each of which employs different chemical agents to break down the polymer chain [22,23]. Glycolysis stands as a promising method for recycling PET. It involves breaking down PET using ethylene glycol (EG) and is more energy-efficient and environmentally friendly (<1 atm, <190 °C) [24,25,26,27,28] than other chemical recycling methods, such as hydrolysis (10–40 atm, 200–300 °C) [29,30] and pyrolysis (1–4 atm, 400–700 °C) [31], which often demand more stringent operational conditions. Moreover, it results in an effective depolymerization of PET into useful molecules like bis(2-hydroxyethyl) terephthalate (BHET). However, as with other chemical recycling approaches, issues due to the possible presence of contaminants in PET can affect the purity of the resulting monomers. Finally, EG is not only inexpensive and widely available, but additionally, it can also be produced from renewable resources, aligning with green chemistry principles, and reducing reliance on fossil fuels [32,33].

The complete PET glycolysis process involves (i) the diffusion of EG into the bulk of PET particles; (ii) the attack on amorphous regions, leading to the generation of oligomers; (iii) the further production of oligomers and a few BHET molecules in the amorphous regions, still involving distinct solid and liquid phases; (iv) the fragmentation into primarily crystalline small oligomer entities; (v) the solubilization of oligomers into the liquid phase; and (vi) the generation of BHET molecules [34]. The obtained BHET molecules can be reused to produce new PET, thereby supporting a closed-loop recycling system.

This process is typically aided by catalysts, co-solvents, supercritical conditions, or radiation-assisted methods [22,35,36], among other methods, which enhance transesterification reactions by breaking ester bonds and favoring hydroxyl group termination. Catalysts for PET glycolysis, such as metal salts, metal oxides [25,37], and zeolites [34,38], have garnered significant attention. However, concerns about environmental impact, technical issues, and product recovery arise due to the use of these catalysts. They can introduce toxicity and disposal challenges, with potential environmental contamination from heavy metals or other harmful substances. Technically, catalysts may suffer from deactivation, high costs, and issues with selectivity, affecting process efficiency. Additionally, product recovery can be complicated by catalyst residues, impacting the purity and yield of the recovered monomers. Therefore, alternative catalysis strategies, like the use of neoteric solvents, have been explored. These neoteric solvents, which include ionic liquids, deep eutectic solvents, and supercritical fluids, offer innovative approaches for the chemical recycling of PET, and can selectively dissolve PET at lower temperatures and pressures compared to traditional methods, enhancing efficiency and reducing environmental impact [39,40]. Among them, ionic liquids (ILs) have been explored for advanced recycling and upcycling [41]. ILs, entirely composed of ions, are liquid at room temperature and possess high thermal stability, strong solvation for various compounds, good electrochemical stability, non-volatility, and low flammability [42]. ILs are generally recyclable and reusable, which adheres to circular economy principles [41,43,44,45,46]. These properties position ILs as promising catalytic co-solvents for scaling up plastic waste valorization [47,48].

In PET chemical recycling, ILs have shown potential for efficient depolymerization under mild conditions, with an easier recovery of both products and ILs. They can be used alone [45], or combined with water [49], ethanol [50], or EG [45,46,49,50,51,52,53,54,55] to promote PET hydrolysis, ethanolysis, or glycolysis, respectively.

Metal-free ionic liquids (MFILs) are a subclass of ionic liquids that do not contain metal cations as part of their chemical composition. These liquids entirely consist of non-metallic organic cations, paired with organic or inorganic anions. They represent a versatile and promising class of solvents with diverse applications across various fields of chemistry and industry. Their customizable nature and environmentally friendly characteristics make them valuable alternatives to traditional solvents and metal-containing ionic liquids. The possibility to combine different cations with different anions allows for the customization of MFILs for a specific application and conditions, enhancing the process efficiency. In addition, their easy recovery and thermal stability make possible their reuse in some cycles, which results in an economic and sustainable benefit.

Among MFILS, 1-ethyl-3-methylimidazolium acetate [Emim] and 1-butyl-3-methylimidazolium [Bmim] can be classified as aprotic ionic liquids (APILs), often paired with various anions, such as chloride [Cl] or acetate [OAc], known for their unique properties and applications as a versatile class of solvents and reaction media with broad applicability, tunable properties, environmental friendliness, and functional diversity [56,57]. In the chemical recycling context, it has been proposed that the hydrogen of the cationic species forms a hydrogen bond with the carbonyl oxygen (C=O) of the ester group, increasing the electron density of the carbon atom. This carbon is then attacked by the oxygen atom of the solvent, such as water or alcohol, leading to the breakdown of the macromolecule into its primary components [58].

Previous studies on the use of metal-free [Emim]- or [Bmim]-based aprotic ionic liquids (APILs) for chemical recycling are summarized in Table 1. Generally, these APILs have been applied to the recycling of polyesters such as poly(ethylene terephthalate) (PET) and polylactide (PLA), as well as polyamide (PA6) and poly-(3-hydroxybutyrate) (PHB), in combination with different solvents to perform glycolysis, hydrolysis, or methanolysis. The type of APIL, the mass proportion of polymer to ionic liquid and solvent, along with the temperature and reaction time, were demonstrated to have a crucial role in all cases. In the glycolysis of PET, results report complete polymer conversion to bis(2-hydroxyethyl) terephthalate (BHET), with yields ranging from 55% to 85%. However, few studies apply a systematic experimental approach based on statistical factorial analysis. The factorial method in the design of experiments offers significant advantages, such as the comprehensive analysis of multiple factors and their interactions, with the possibility of identifying and quantifying interaction effects, along with the robustness of the statistical approach.

Altogether, this study aims to explore the effectiveness of the neoteric [Emim][OAc] and [Bmim][OAc] aprotic ionic liquids as catalytic co-solvents in the glycolysis reaction of PET with ethylene glycol (EG). The research focuses on understanding the reaction thermal kinetics of the glycolytic process and optimizing the conversion of PET by examining key process factors, including temperature, plastic-to-ionic liquid mass ratio, and plastic-to-solvent mass ratio.

## 2. Materials and Methods

### 2.1. Materials and Reagents

Poly(ethylene terephthalate) (PET) pellets, grade SEDAPET SP04, were supplied by Catalana de Polimers S.A., Grup LaSeda (Barcelona, Spain) as bottle-grade PET, with a size of 2–3 mm in diameter and an intrinsic viscosity of 0.80 dL·g^−1^. Ethylene glycol (EG) (>99.0%) was provided by VWR Chemicals (Radnor, PA, USA). 1-ethyl-3-methylimidazolium acetate [Emim][OAc] > 98% and 1-butyl-3-methylimidazolium acetate [Bmim][OAc] > 95% were supplied by Iolitec (Heilbronn, Germany).

### 2.2. Chemical Recycling of PET

A total of 2 g of the PET pellets were introduced into 40 mL glass vials with threaded plugs. The reaction media was composed of different mass proportions of EG and IL, as shown in Table 2. The vials were placed into an isolated Teflon^®^-based setup and subsequently located onto an Onilab MS7-H550-Pro magnetic hot plate (Barcelona, Spain). Glycolysis was carried out at fixed temperatures ranging between 160 and 190 °C (±0.1 °C), under magnetic stirring at 850 rpm (±1 rpm), at different times up to 120 min. Afterward, the reaction was stopped by placing the vials in a fridge at 4 °C for 15 min, and the remnant solid PET was separated by vacuum filtration and dried at 55 °C in an Argolab TCN-30 Plus convection oven (Carpi, Italy) until constant mass was achieved. The liquid solution was kept at 4 °C for 24 h to achieve the precipitation of the glycolysis product (GP), which was then vacuum-filtered, washed with ultra-pure water, and dried in the above-mentioned convection oven at 55 °C until constant mass was achieved. The PET conversion (*X_PET_*) was calculated according to Equation (1), assuming that the GP mainly involved bis(2-hydroxyethyl) terephthalate (BHET), as further confirmed.

(1) $$X_{PET}\;(\%)=\left(\frac{m_{0\;PET}-m_{f\;PET}}{m_{0\;PET}}\right)\times 100$$

where *m*_0_ *_PET_* and *m_f PET_* are the initial and final masses of PET (g), respectively.

### 2.3. Statistical Design of Experiments

The maximization of the PET conversion (*X_PET_*) was carried out with a comprehensive statistical strategy, involving the evaluation of the following selected parameters: (i) temperature T (170, 180, and 190 °C), (ii) plastic-to-ionic liquid mass ratio P/IL (4:1, 2:1 and 1:1, PET:IL, m:m), and (iii) plastic-to-solvent mass ratio P/S (1:2.5, 1:5 and 1:7.5, PET:EG, m:m), following a Box–Behnken design (BBD) with the factors/levels indicated in Table 2, together with the factual mass of EG and IL used in the experiments, for 120 min of reaction. The ionic liquid-to-solvent mass ratio therefore ranged from 1:7.5 to 1:10. A detailed list of the experiments is shown in Supplementary Material. Statistical analyses were carried out with the aid of Minitab 19.1 (State College, PA, USA).

### 2.4. Analytical Techniques

The chemical structure of BHET was evaluated through Fourier-Transform infrared spectroscopy (FTIR) in an Agilent Cary 630 spectrometer (Santa Clara, CA, USA). Spectra were collected by the attenuated total reflectance (ATR) mode in the wavenumber range between 600 to 4000 cm^−1^, with a resolution of 4 cm^−1^ during 64 accumulations.

Thermogravimetric analysis (TGA) was conducted using a Perkin Elmer TGA 7 device (Waltham, MA, USA) with samples of 4 mg into 70 µL perforated alumina crucibles. Samples were heated from 25 to 800 °C with a rate of 10 °C·min^−1^ under argon atmosphere with a gas flux of 50 mL·min^−1^.

Differential scanning calorimetry (DSC) was carried out in a Mettler-Toledo DSC 820e setup (Columbus, OH, USA). A total of 4 mg per sample were introduced into 70 µL Mettler-Toledo perforated alumina crucibles, and samples were heated from 30 up to 280 °C with a rate of 10 °C·min^−1^ under nitrogen atmosphere with a gas flux of 50 mL·min^−1^.

### 2.5. Thermal Kinetic Analysis of the Glycolysis of PET

A kinetic analysis was conducted as a useful approach to evaluate the feasibility of the glycolysis catalyzed by [Bmim][OAc] and [Emim][OAc] for PET depolymerization and its potential scaling. For this study, 2 g of PET pellets were treated with 5 g of EG (P/S = 1:2.5) and 2 g of IL (P/IL = 1:1) using both [Bmim][OAc] and [Emim][OAc].

The reaction rate of the PET glycolysis was assumed to be controlled by a first-order kinetic equation, as some authors have reported high correlations using this model for chemical PET degradation [45,52,63,64], according to Equation (2).

(2) $$\frac{d\left(C_{PET}\right)}{dt}=-k\times C_{PET}$$

where *C_PET_* (mol·L^−1^) is the PET concentration in the reaction media, *t* (min) is the reaction time, and *k* (min^−1^) is the kinetic constant. *C_PET_* was determined based on the PET conversion using Equation (3).

(3) $$C_{PET}=C_{PET,t=0}\left(1-\frac{X_{PET}}{100}\right)$$

Finally, Equations (2) and (3) were substituted, rearranged, and integrated over time, resulting in Equation (4).

(4) $$\ln\frac{1}{1-\frac{X_{PET}}{100}}=k\times t$$

in which $\ln\frac{1}{1-\frac{X_{PET}}{100}}$ represents the reaction rate.

## 3. Results and Discussion

The glycolysis reaction of PET using [Bmim][OAc] and [Emim][OAc] as catalytic co-solvents was examined from multiple perspectives to assess its suitability for a waste-to-gate circular model. The waste-to-gate circular model converts waste materials into valuable products, reintegrating them into the production cycle to minimize waste and resource consumption. This approach supports a closed-loop system, promoting sustainability and resource efficiency. Therefore, the reaction’s efficacy in converting PET to BHET was firstly validated, according to the glycolysis reaction schematized in the Supplementary Material [45]. Next, thermal kinetics were analyzed to compare the energy requirements of this reaction with those reported in the literature. Afterward, a statistical design of experiments was then conducted to determine the relative influence and interdependence of the key parameters governing the glycolytic reaction, i.e., temperature, PET-to-solvent mass ratio (P/S), and PET-to-ionic liquid mass ratio (P/IL). Finally, an optimization procedure was performed to establish the optimal values for these parameters to maximize PET conversion.

### 3.1. Glycolysis of PET to Obtain BHET

**Figure 1:**
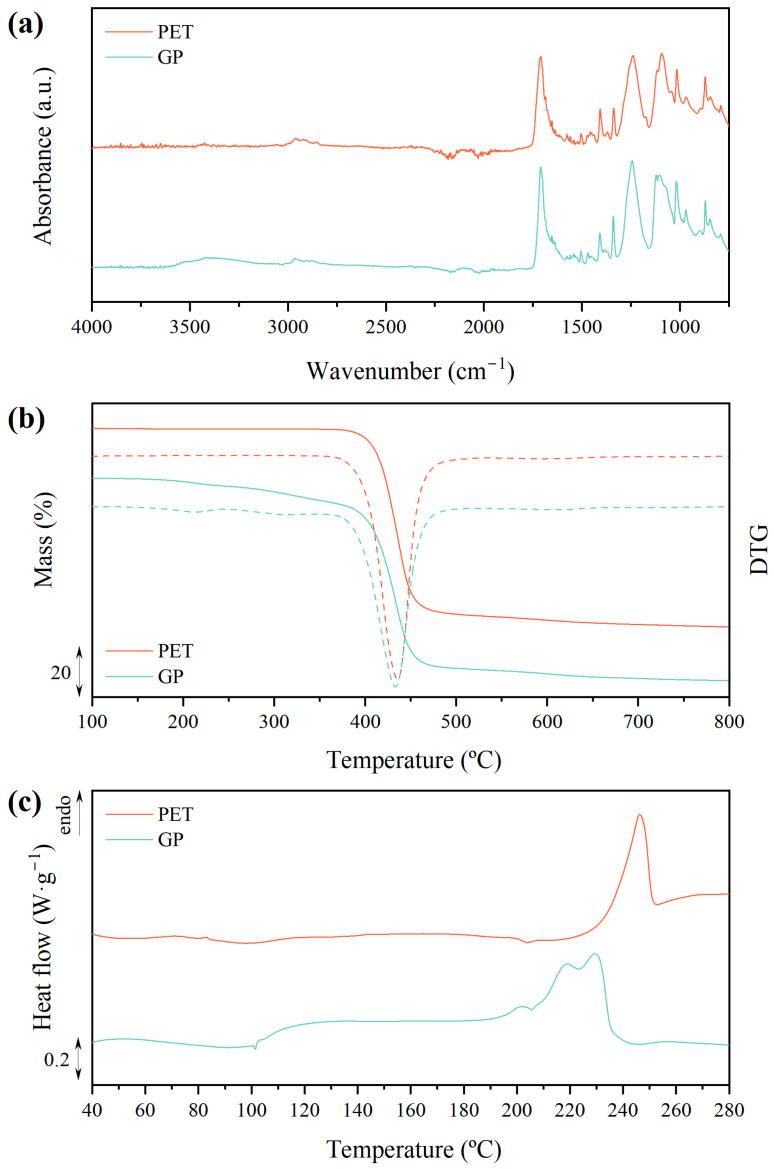
Spectroscopic and thermal characterization of PET and glycolysis product: (**a**) Infrared spectra; (**b**) thermogravimetric (solid) and derivative thermogravimetric curves (DTG) (dash); (**c**) first heating calorimetric scan.

The glycolysis product (GP) was qualitatively assessed after the precipitation–washing–drying sequence to verify the effective PET chain scission and the generation of bis(2-hydroxyethyl) terephthalate (BHET). The comparison between the spectroscopic and thermal characteristics of the GP and raw PET is shown in Figure 1. In the infrared spectra shown in Figure 1a, new bands in the 3600–3300 cm^−1^ range indicated the O-H stretching of both free and intermolecular bonded units. A broad band at 3200–2700 cm^−1^ was attributed to O-H stretching, signaling the formation of -OH terminated species during glycolysis. The 1710 cm^−1^ band, linked to C=O stretching from carbonyl groups, was more intense in the GP, with additional prominent peaks at 1339 cm^−1^ due to O-H bending, and 1245 cm^−1^ and 1103 cm^−1^ ascribed to the C-O stretching of aromatic esters and primary alcohols, respectively. These features are indicative of glycolyzed oligomers and monomers, typical of PET degradation via glycolysis. The presence of BHET was confirmed by the 1518 cm^−1^ band, corresponding to the aromatic phenyl ring of BHET, and a 3280 cm^−1^ band overlapping with the O-H stretching band.

The thermogravimetric analysis results shown in Figure 1b revealed that raw PET remained stable up to 375 °C, whereas the GP began to lose mass at around 160 °C, with a ~14% mass loss peaking at 209 °C. This early mass loss is due to the volatilization of low molar mass compounds, such as BHET, occurring alongside repolymerization processes. Decomposition continued above 400 °C, a typical temperature for PET. The literature shows that BHET undergoes thermal decomposition with an initial mass loss (20–50%) above 200 °C, followed by a second mass loss (50–75%) around 450 °C. Despite the lower initial mass loss proportion compared to reported values, the significant presence of BHET in the GP was inferred.

In the calorimetric thermograms plotted in Figure 1c, the endothermic melting transition for PET, peaking at 246 °C, corresponds to a crystallinity degree (*X_c_*) of ~20%, aligning with previous data for semicrystalline PET. For the GP, a wide endothermic process started above 100 °C, followed by a minor endothermic peak at 201 °C, and a main transition with a bimodal melting pattern at 218 °C and 228 °C. Although this main event resembles PET’s melting transition, previous thermogravimetric data indicate that the GP begins thermal decomposition below these temperatures. Thus, this endothermic event likely represents the partial thermal decomposition of the GP. Studies have identified the melting peak of pure BHET at around 110 °C, and for dimers/oligomers formed during glycolysis, it is above 170 °C according to the obtained results in this work. In summary, the glycolysis of PET mainly yielded BHET, which may coexist in equilibrium with dimers, trimers, and small oligomers. BHET crystals are mainly present in alpha modes, usually together with delta modes, despite the fact that beta and gamma configurations are also physically likely [65]. Further research on the identification of the polymorphs is relevant for down-streaming the process towards separation, since the nature and shapes of the crystals influence operations such as filtration, washing, or drying, which are crucial for upscaling the process, as detailed in the literature [66]. 

### 3.2. Thermal Kinetics of the Glycolysis of PET Using [Emim][OAc] and [Bmim][OAc] as Catalytic Co-Solvents

**Figure 2:**
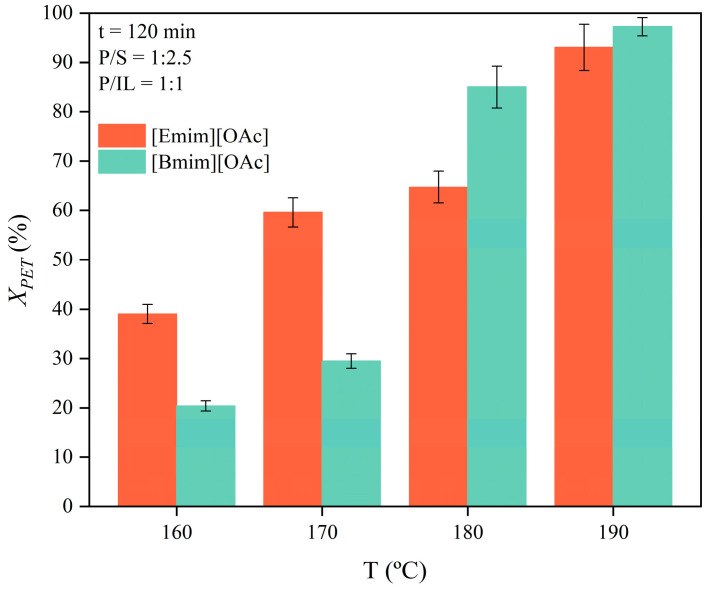
Effect of temperature on the glycolytic conversion of PET using [Emim][OAc] and [Bmim][OAc]. Experimental conditions: P/S = 1:2.5 (m:m); P/IL = 1:1 (m:m); t = 120 min.

Assessing the thermal kinetics is essential for determining the energy requirements and feasibility of scaling up the IL-driven catalyzed glycolysis of PET. The effect of the reaction temperature, ranging from 160 to 190 °C, at P/S = 1:2.5 and P/IL = 1:1, on the conversion of PET is shown in Figure 2. The reader must note that experiments without the presence of the ionic liquids [Emim][OAc] and [Bmim][OAc] in the reactor were not significant, with conversions lower than 3 %.

A higher depolymerization rate was observed as temperature increased, according to the endothermic behavior of the reaction. At 120 min, the PET conversion increased from 20.4% to 29.5% at 160 °C and 170 °C, respectively, whilst it reached values of 85.0% and 97.2% at 180 °C and 190 °C, respectively. These results suggest that temperatures above 180 °C were significantly more beneficial in improving the PET glycolysis reaction when [Emim][OAc] and [Bmim][OAc] were applied as catalytic co-solvents. Regarding the effect of the IL on the reaction rate, different behaviors were observed at low and high temperatures. At temperatures above 180 °C, the use of [Bmim][OAc] led to a higher conversion, but it resulted in lower conversions below 180 °C. An opposite trend was observed when using [Emim][OAc]. Altogether, it was found that the used ILs presented a different catalytic activity as a function of temperature.

**Figure 3:**
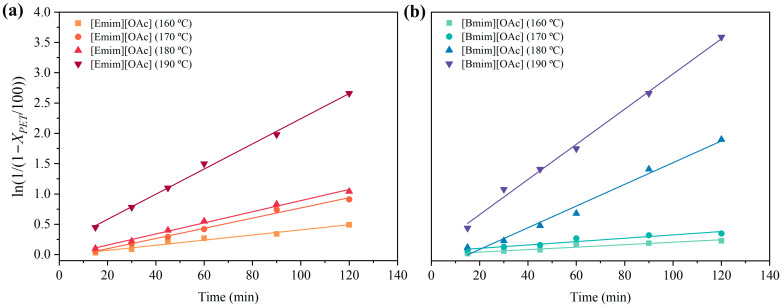
Conversion rate of PET as a function of temperature using (**a**) [Emim][OAc] and (**b**) [Bmim][OAc] as catalysts. Experimental conditions: P/S = 1:2.5 (m:m); P/IL 1:1 (m:m). Note that error bars (~2–5%) were omitted for the sake of clarity.

The reaction rate was expressed according to Equation (4), assuming that PET glycolysis is controlled by a first-order kinetic model, as previously discussed in the literature [52,63,64]. The reaction rate evolution as a function of time for the different temperatures is shown in Figure 3. The results were fitted employing a linear regression for both [Emim][OAc] and [Bmim][OAc] to determine the kinetic coefficients, gathered in Table 3. The obtained linear correlative coefficients (*R*^2^) above 0.97 indicated that the PET glycolysis catalyzed with the tested IL agreed with the proposed first-order kinetic model. Nevertheless, for comparison purposes, a kinetic analysis based on the shrinking-core model proposed by other authors was also evaluated, and the results are shown in the Supplementary Material. However, the linear correlative coefficients were significantly lower, corroborating that the catalyzed glycolysis of PET pellets in this work followed the first-order model.

In line with previous observations for conversion, at temperatures below 180 °C, higher kinetic coefficients were obtained when using [Emim][OAc], with values between 4.01 × 10^−3^ and 7.51 × 10^−3^ min^−1^. However, [Bmim][OAc] led to higher kinetic coefficients at temperatures above 180 °C, with values ranging from 14.55 × 10^−3^ and 29.98 × 10^−3^ min^−1^. Altogether, the PET depolymerization rate depended only on the PET concentration in the reaction media at the reaction condition tested, reaching PET conversion values near 100% at 120 min. Thus, the upscaling of this technology, as well as the process control at a large scale, could be presented as feasible, and contribute to progress in the field of the chemical recycling of PET.

**Figure 4:**
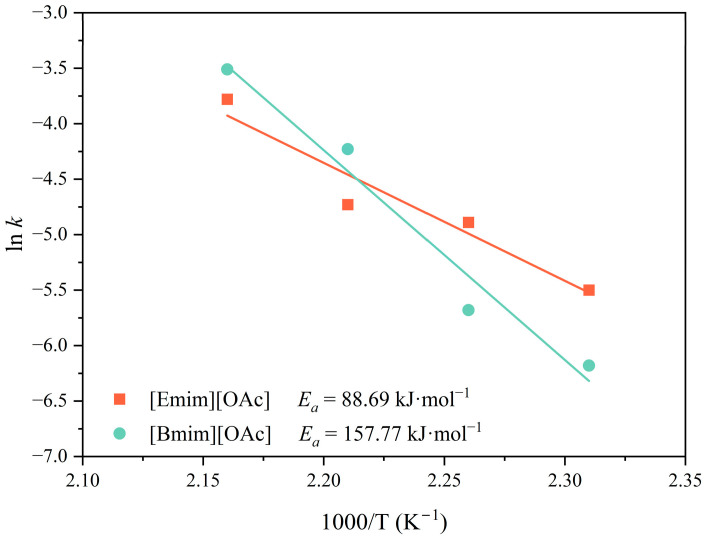
Arrhenius plot of the kinetic constant of the PET for both ionic liquids [Emim][OAc] and [Bmim][OAc]. Note that error bars between 2 and 5% were omitted for the sake of clarity.

In order to determine the apparent activation energy (*E_a_*, kJ·mol^−1^) of the PET glycolytic depolymerization, the dependency of the kinetic coefficients with temperature was elucidated using the Arrhenius equation in its linearized form (Equation (5)).

(5) $$\ln k=\ln A-\frac{E_{a}\times 1000}{R\times \left(T+273.15\right)}$$

where *A* (min^−1^) is the pre-exponential factor, *R* (8.314 J K^−1^ mol^−1^) is the ideal gas constant, and *T* (°C) is the reaction temperature. The Arrhenius plots for both IL [Emim][OAc] and [Bmim][OAc]-driven kinetics are shown in Figure 4, where lineal correlation coefficients above 0.93 indicated that the kinetic coefficient presented an exponential dependency with temperature. Pre-exponential factors of 1.91 × 10^16^ and 1.98 × 10^8^ min^−1^ and activation energies of 88.69 and 157.77 kJ·mol^−1^ were obtained when using [Emim][OAc] and [Bmim][OAc], respectively. It should be highlighted that the obtained *E_a_* values were significantly different even though both ILs presented a similar nature. This different performance of *E_a_* could suggest that [Emim][OAc] led to more beneficial interactions involved in the reaction mechanism, increasing its catalytic activity. When using ILs, most of the proposed mechanisms start with the interaction between the IL cation and the oxygen of the C=O ester bond of PET, which increases the electrophilicity of the carbon, and the formation of a hydrogen bond between the OH group of the EG and IL anion, which increases the electronegativity of the hydroxyl oxygen [52,67]. Then, the formed carbon cation is attacked by this hydroxyl oxygen through a nucleophilic reaction, resulting in the chain scission of PET. Hence, the lower *E_a_* of [Emim][OAc] could be explained by the shorter radical chain of the ethyl group in [Emim]^+^, which makes it more electrophilic than [Bmim]^+^, becoming more active to protonate the carbon of the ester group.

**Figure 5:**
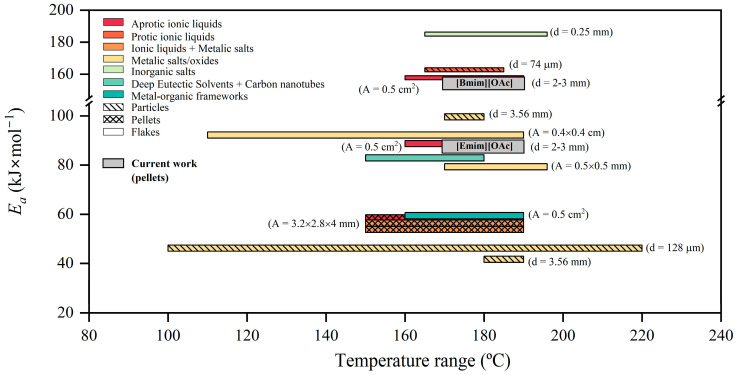
Activation energy (*E_a_*) obtained from kinetic analyses of the heterogeneous glycolysis of PET in the solid state reported in the literature as a function of different catalysts and reaction conditions.

Other works in the literature assessing the heterogeneous glycolysis of PET in a solid state found comparable activation energies, as shown in Figure 5. Particularly, the *E_a_* obtained for [Bmim][OAc] (157.78 kJ·mol^−1^) was more comparable to those obtained for the ILs choline acetate [Ch][OAc] and 1,5-diazabicyclo-5-nonene m-cresol [DBN][m-cresol], with reported values of 131.3 and 162.8 kJ·mol^−1^, respectively [67,68]. It should be mentioned that these two works treated small PET particles with a diameter ≤ 400 µm and applied a shrinking-core model, showing lineal correlative coefficients over 0.98. On the other hand, Wang et al. evaluated the IL [Bmim][Cl] with the same cation as that tested in the present work, reporting an *E_a_* value of 232.8 kJ·mol^−1^ [45]. This high *E_a_* value was in line with the less catalytic activity reported for ILs containing halogen anions such as [Cl] and [Br] [53]. Al-Sabagh et al. obtained *E_a_* values of 58.5 kJ·mol^−1^ when using [Bmim][OAc] as a catalyst for the glycolysis of PET in pellet form at similar reaction conditions to the present work [52]. Differences in *E_a_* values at similar experimental conditions could be attributed to the different nature of PET and the size or shape of the particles. Even though the particle shape and size are closely related to the available surface area, the diffusion performance of reactants, or the thermal conductivity [63], both studies used pellets with pellet sizes of 2–3 mm in diameter, so that differences cannot be attributed to the particle shape or dimensions. Nevertheless, the molar mass of the raw material is another crucial factor. While Al-Sabagh et al. reported values for the intrinsic viscosity of 0.64 dL·g^−1^, the commercial PET resin grade used in the current study has an intrinsic viscosity of 0.80 dL·g^−1^, corresponding to a greater molecular weight. Therefore, although molar mass is not the only determining factor, it influences the activation energy of the chemical depolymerization of a polymer due to its impact on the physical structure, crystallinity, and intermolecular properties of the material and subsequently on the diffusivity of the glycolytic media.

Regarding other types of catalysts, metal salts and their combination with ILs led to lower *E_a_* values than applying inorganic salts, metal oxides, and ILs with values as low as ~40 kJ·mol^−1^. However, the use of metal-based catalysts involves some limitations for the implementation at a large scale regarding their high cost, negative environmental impact, and low biocompatibility [67]. Finally, *E_a_* values of 59.4 and 82.9 kJ·mol^−1^ have been reported when c-ZIF-8@SiO_2_ nanoparticles were applied together with photothermal catalysis and solar-thermal catalysis in combination with [Ch]_3_[PO_4_] and CNT-PDA (multi-walled carbon nanotubes pre-modified by polydopamine), respectively, even though these technologies are in an early stage of development. As a final remark, using metal-free ILs such as [Emim][OAc] for the catalyzed glycolysis of PET was demonstrated to achieve mild energetic demands, comparable to other studies in the literature.

### 3.3. Individual and Interaction Effects on the Glycolysis of PET

The influence of different parameters in the glycolysis of the PET reaction using [Bmim][OAc] and [Emim][OAc] as catalytic co-solvents was studied to achieve the conditions that allow for the obtainment of the maximum conversion and yield. The parameters evaluated for each IL were the temperature (T), the plastic-to-solvent mass ratio (P/S), and the plastic-to-IL mass ratio (P/IL) on an individual basis and in different combinations. The use of statistical factorial methods is of high importance in finding the optimal processing parameters [64,69,70,71,72]. Following a Box–Behnken design, the evaluated factors were (i) the T (170, 180, and 190 °C), (ii) the P/S (1:2.5, 1:5 and 1:7.5; PET:EG, m:m), and (iii) the P/IL (4:1, 2:1 and 1:1, PET:IL, m:m). A regression model for a Surface Response Model was applied, as shown in Equation (6).

(6) $$y=\alpha_{0}+\sum \alpha_{i}x_{i}+\sum_{i}\alpha_{ii}x_{i}^{2}+\sum_{i<j}\sum_{j}\alpha_{ij}x_{i}x_{j}$$

where *y* is the expected response (conversion), *α*_0_ is a constant, and *α_i_*, *α_ii_*, and *α_ij_* are linear (*α_i_* = *α_T_*, *α_P/S_*, *α_P/IL_*), quadratic (*α_ii_* = *α_T×T_*, *α_P/S×P/S_*, *α_P/IL×P/IL_*), and interaction (*α_ij_* = *α_T×P/IL_*, *α_T×P/S_*, *α_P/S×P/IL_*) coefficients between the *x_i_* and *x_j_* factors (*x* = T, P/S, P/IL), respectively. The method of least squares regression was used to calculate the polynomial coefficients, which was validated through the analysis of variance (ANOVA). The relevance and significance of each coefficient in the equations were characterized by the Fischer–Snedecor *F*-test value and *p*-values, respectively, considering a significant effect when the *p*-value was lower than 0.05 at a 95% confidence level. The coefficients with significant contributions to the conversion were finally included in Equations (7) and (8) as models for the [Emim][OAc] and [Bmim][OAc], respectively. Quadratic parameters were not statistically significant and did not contribute to the response model.

(7) $$X_{{PET}_{\left[Emim\right]}}\left(\%\right)=0.209\times T-84.3\times P/IL-28.21\times P/S+0.590\times P/IL\times T+0.151\times T\times P/S$$

(8) $$X_{{PET}_{\left[Bmim\right]}}\left(\%\right)=0.234\times T-160.8\times P/IL-35.75\times P/S+0.984\times P/IL\times T+0.192\times T\times P/S$$

**Figure 6:**
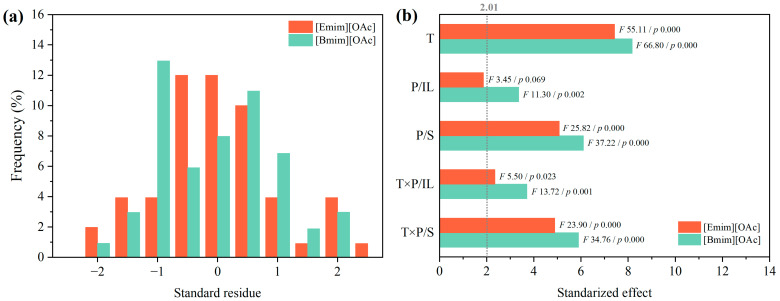
(**a**) Histogram for the standard residue for both ionic liquids [Emim][OAc] and [Bmim][OAc]; (**b**) Pareto plot of the significant effects on the conversion reaction of glycolysis of PET. Significance level = 2.03. t = 120 min. *F* stands for the Fischer–Snedecor *F*-value, and *p* stands for the *p*-value of statistical significance of analysis of variance (ANOVA).

Figure 6 shows the statistical analysis of variance obtained with the Pareto chart, together with the histogram of the standard residue. On the one hand, the model’s suitability was evaluated by analyzing the residuals’ normal and random distribution concerning the fitted values, as depicted in Figure 6a. The standardized residuals were all contained within the [−2, 2] range, indicating a 95.5% confidence level in the results. Adjusted regression coefficients of 0.9624 and 0.9595 were obtained for the responses of the chemical recycling of PET driven by [Emim][OAc] and [Bmim][OAc], respectively, confirming the model’s reliability within the experimental limits of {T, P/S, and P/IL}. On the other hand, Figure 6b represents the Pareto chart, with an indication of the Fischer–Snedecor *F*-value and significance *p*-value, for the significant (i.e., *p*-value < 0.05) linear and interaction parameters, as obtained from the statistical analysis of variance. In this plot, the higher the *F*-value, the greater the impact of the factor on the response.

Regardless of the IL type, the linear effects of temperature (T) and the plastic-to-solvent mass ratio (P/S) were the most significant. Following this, the interaction of temperature and P/S played a relevant role in the conversion of PET. Even though the effect of the plastic-to-ionic liquid mass ratio (P/IL) in combination with temperature was significant, a minor effect was found for the contribution of P/IL. Other quadratic (T × T, P/IL × P/IL and P/S × P/S) and interaction effects (P/S × P/IL) were not statistically significant for the PET conversion. In general, it can be highlighted that the effect of linear and interaction factors was slightly more relevant when using [Bmim][OAc] than [Emim][OAc] as catalytic co-solvents, again suggesting a slightly different role on the glycolytic reaction of the used ionic liquids. The relevance sequence for both ionic liquids involved the linear factors T and P/S, followed by the interaction factors T × P/S and T × P/IL, with P/IL being the less significant parameter.

**Figure 7:**
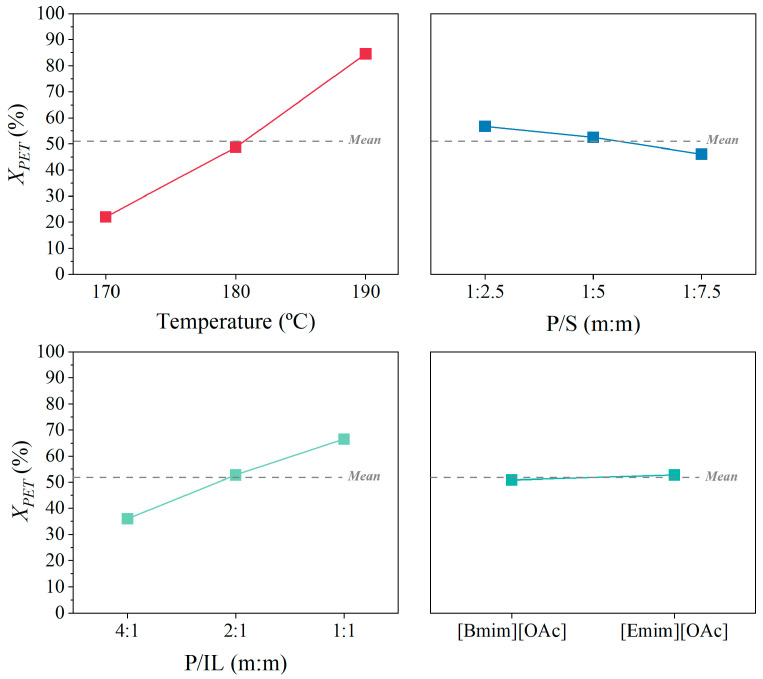
Main effects plot of the factors temperature, P/IL, P/S, and IL type on the PET conversion (*X_PET_*).

**Figure 8:**
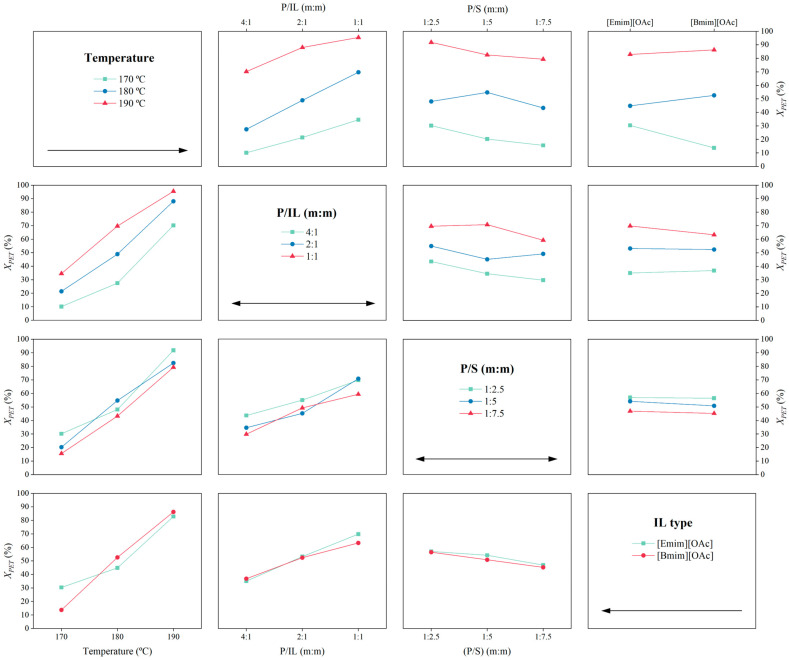
Interaction effects plot of the factors temperature, P/IL, P/S, and IL type on the PET conversion (*X_PET_*).

To visualize the impact of the different parameters individually involved in the glycolysis of the PET reaction (T, P/S, P/IL, and IL type), the main effects (MEF) plot and the interaction effects (IEF) plot are shown in Figure 7 and Figure 8.

The MEF plot in Figure 7 shows the average of the experiments performed at a single condition, where the horizontal line around ~52% represents the average conversion for all the experiments performed in this work (grand mean). This helps evaluate the statistical significance of the parameters and their impact on the response. On the one hand, the PET conversion was significantly enhanced with the temperature, achieving a rise of 20% to 90% from 170 °C to 190 °C. This indicates that higher temperatures accelerate the glycolytic process, leading to the more efficient depolymerization of PET. On the other hand, the plastic-to-solvent mass proportion (P/S) revealed a less significant effect, where a difference lower than 10% was observed between a relation of 1:2.5 and 1:1.75. The results show that less solvent enhanced the glycolytic process, implying that excess solvent may dilute the reactants and slow down the reaction. Regarding the plastic-to-ionic liquid mass proportion (P/IL), a higher presence of IL allowed us to achieve a higher conversion, moving from 35 to 67% at P/IL of 4:1 and 1:1, respectively. This suggests that the ionic liquid plays a crucial role as a catalytic co-solvent for facilitating the glycolytic process. Finally, there was not a significant effect between the use of [Emim][OAc] or [Bmim][OAc], where the conversion in all cases virtually coincided with the grand mean.

The interaction effects (IEF) diagram shown in Figure 8 represents the interaction of T × P/S, P/S × P/IL, T × P/IL, and the combination of T, P/S, and P/IL with the IL type. This matrix-shaped plot aids in visualizing both the statistical significance of these interactions and their influence on the response. Again, it was demonstrated that the temperature played a crucial role in the glycolysis of PET with [Emim][OAc] or [Bmim][OAc]. The interaction of temperature with the other factors always resulted in a greater PET conversion; T × P/IL was the combination with a critical improvement when temperature increased. As for the interaction of plastic-to-ionic liquid mass ratio (P/IL) with other factors, the most relevant collaboration was with temperature, as previously mentioned, and the other combinations were non-relevant in terms of the PET conversion. Furthermore, the plastic-to-solvent mass ratio (P/S) revealed a significant influence in combination with temperature. In general, a lower P/S ratio with a higher P/IL proportion enhanced the PET conversion. Finally, in terms of IL type, the interaction with temperature was also relevant, with better performance for the [Emim][OAc] at lower temperatures and the [Bmim][OAc] at higher temperatures.

### 3.4. Maximization of Glycolysis of PET Using [Bmim][OAc] and [Emim][OAc] as Catalytic Co-Solvents

Once the individual and combined effects of the different parameters and their significance on the PET conversion were considered, the maximization results in this section involved the proposal of different solutions for the combination of temperature, P/IL, and P/S conditions to enhance the glycolytic depolymerization of PET. From a performance perspective, the higher temperature of 190 °C was selected, as this temperature showed a greater conversion. Moreover, in an energy efficiency scenario, optimal conditions at 180 °C were complementarily explored. The results are gathered in Table 4, where the selected optimal solutions at low and high temperatures are highlighted.

On the one hand, for the temperature of 190 °C, optimal conditions involved the P/IL of 1:1 and the P/S of 1:2.5, which allowed for an optimal predicted conversion of 100% regardless of the use of [Emim][OAc] and [Bmim][OAc]. Given the critical role of the IL in the reaction, a higher IL proportion was selected. However, the lowest solvent percentage was chosen. Although significant quantities of IL are needed to maximize the conversion, numerous studies in the literature have assessed the successful reusability of [Emim][OAc] and [Bmim][OAc] [73,74,75]. This reusability is crucial both from economic and environmental perspectives for the evaluated system.

On the other hand, at 180 °C, optimal conditions were found at P/IL of 1:1 and P/S of 1:5, with predicted conversions of 80% and 78% with [Emim][OAc] and [Bmim][OAc], respectively. As can be observed, the solvent proportion was necessarily increased, in comparison to the optimal conditions found at 190 °C. In this context, achieving a sustainable chemical recycling strategy for the valorization of PET requires carefully balancing several factors. Firstly, energy demand must be optimized to ensure the process is both economically viable and environmentally friendly. Secondly, the solvent and ionic liquid consumption should be minimized without compromising the efficiency of the conversion process. This involves selecting reagents that are not only effective but also reusable and less harmful to the environment.

## 4. Conclusions

This study successfully demonstrated the effectiveness of the neoteric imidazolium-based ionic liquids [Emim][OAc] and [Bmim][OAc] as catalytic co-solvents of the glycolysis of PET with ethylene glycol (EG). The detailed investigation into the reaction thermal kinetics revealed that both ionic liquids significantly enhanced the depolymerization rate of PET compared to traditional methods, being more energy-efficient with the contribution of [Emim][OAc] with an activation energy of 88.69 kJ·mol^−1^, which was explained by a first-order model. The effect of the key process parameters, including temperature (T), plastic-to-ionic liquid mass ratio (P/IL), and plastic-to-solvent mass ratio (P/S), was evaluated by means of an analysis of variance (ANOVA), following a statistical Box–Behnken design of experiments, and optimized to achieve the maximum PET conversion. The relevance sequence for both ionic liquids involved the linear factors T and P/S, followed by the interaction factors T × P/S and T × P/IL, with P/IL being the less significant parameter. Optimal conditions involved the P/IL of 1:1 and the P/S of 1:2.5 at 190 °C, which allowed for a predicted conversion of 100% regardless of the use of [Emim][OAc] and [Bmim][OAc]. By finding the optimal balance among these elements, a chemical recycling strategy that is both sustainable and scalable is proposed, contributing to the reduction in PET waste and the promotion of a circular economy within waste-to-gate pathways.

## Figures and Tables

**Table 1:** Previous studies on the use of [Emim] or [Bmim]-based aprotic ionic liquids, with specific anionic species and/or used solvents for the chemical recycling of polymers.

Polymer	Cationic Species	Anionic Species	Solvent	Ref.
Poly(ethylene terephthalate) (PET)	[Emim]^+^	[Terephthalic acid]^−^	Ethylene glycol	[44]
Polyamide 6 (PA6)	[Emim]^+^	[BF_4_]^−^	-	[59]
Poly(3-hydroxybutyrate) (PHB)	[Emim]^+^	[OAc]^−^	-	[60]
Poly(ethylene terephthalate) (PET)	[Bmim]^+^	[OAc]^−^	Ethylene glycol	[52]
Poly(ethylene terephthalate) (PET)	[Bmim]^+^	[Cl]^−^ [HCO_3_]^−^ [Br]^−^ [OH]^−^	Ethylene glycol	[55]
Poly(lactic acid) (PLA)	[Bmim]^+^	[OAc]^−^	Water	[61]
Poly(lactic acid) (PLA)	[Bmim]^+^	[OAc]^−^	Methanol	[62]

**Table 2:** Variables and levels involved in statistical Box–Behnken design of experiments.

Parameter	Variable (Unit)	Coded and Experimental Levels		
		−1	0	1
T	Temperature (°C)	170	180	190
P/IL	PET:IL (m:m) {mass IL} (g)	4:1 {0.5}	2:1 {1}	1:1 {2}
P/S	PET:EG (m:m) {mass EG} (g)	1:2.5 {5}	1:5 {10}	1:7.5 {15}
IL	Ionic liquid type	[Emim][OAc]	-	[Bmim][OAc]

**Table 3:** Kinetic coefficients (*k*) and lineal correlation coefficients (*R*^2^) obtained from the kinetic analysis using the first-order model at different temperatures (T) for both ionic liquids [Emim][OAc] and [Bmim][OAc].

Ionic Liquid	T (°C)	*k* × 10^3^ (min^−1^)	*R* ^2^
[Emim][OAc]	160	4.07	0.9965
	170	7.51	0.9978
	180	8.87	0.9918
	190	22.82	0.9909
[Bmim][OAc]	160	2.07	0.9984
	170	3.40	0.9758
	180	14.56	0.9739
	190	29.98	0.9763

**Table 4:** Maximization results given from the simulations of the regression models. Note: * highlights the selected optimal solutions at low and high temperatures.

	Solution	T (°C)	P/IL (m:m)	P/S (m:m)	*X_PET_* (%)
[Emim][OAc]	*E*_1_	190	2:1	1:2.5	93
	* E_2_		1:1	1:2.5	100
	*E*_3_		1:1	1:5	96
	*E*_4_	180	1:1	1:7.5	61
	*E*_5_		1:1	1:2.5	72
	* E_6_		1:1	1:5	80
[Bmim][OAc]	*B*_1_	190	2:1	1:2.5	95
	* B_2_		1:1	1:2.5	100
	*B*_3_		1:1	1:5	97
	*B*_4_	180	1:1	1:2.5	61
	*B*_5_		1:1	1:7.5	63
	* B_6_		1:1	1:5	78

## Data Availability

Dataset available on request from the authors..

## Supplementary Materials

The following supporting information can be downloaded at: https://www.mdpi.com/article/10.3390/polym16172451/s1, Figure S1: Possible mechanism of the degradation of PET in [bmim]Cl. Reproduced with permission from Hui Wang et al., Green Chemistry; published by Royal Society of Chemistry, 2009; Figure S2: Effect of temperature on the conversion rate of PET using (a) [Bmim][OAc] and (b) [Emim][OAc] as catalysts at different temperatures. Tests conducted with 2, 5 and 2 g of PET, ethylene glycol and ionic liquid, respectively. Kinetic analysis based on the shrinking-core model; Table S1: DOE experimental planning. Note that the table has been ordered for the sake of clarity, but experiments were conducted following an aleatory sequence; Table S2: Kinetic coefficients (*k*) and lineal correlation coefficients (*R*^2^) obtained from the kinetic analysis using the first-order model at different temperatures (T) for both ionic liquids [Bmim][OAc] and [Emim][OAc]. Kinetic analysis based on the shrinking-core model. References [45,67,76] are cited in the supplementary materials.
