# Supplementary material for: Factorial Analysis and Thermal Kinetics of Chemical Recycling of Poly(ethylene terephthalate) Aided by Neoteric Imidazolium-Based Ionic Liquids
Source: Polymers (Basel). 2024 Aug 29;16(17):2451. doi: 10.3390/polym16172451 (PMC11397852; doi:10.3390/polym16172451)
Supplement: Supplementary file 1 [file polymers-16-02451-s001.zip › polymers-3115424-supplementary.pdf]

Supplementary Material

Factorial Analysis and Thermal Kinetics of Chemical Recycling of Poly(ethylene terephthalate) Aided by Neoteric Imidazolium-Based Ionic Liquids

Oscar Gil-Castell [1], Ramón Jiménez-Robles [1], Alejandro Gálvez-Subiela [1], Gorka Marco-Velasco [1], M. Pilar Cumplido [2], Laia Martín-Pérez [1], Amparo Cháfer [1,*] and Jose D. Badia [1,*]

[1] Research Group in Materials Technology and Sustainability (MATS), Department of Chemical Engineering, School of Engineering, Universitat de València, Av. Universitat s/n, 46100 Burjassot, Valencia, Spain; oscar.gil@uv.es (O.G.-C.)

[2] Plastic Technology Centre (AIMPLAS), Gustave Eiffel 4, 46980 Paterna, Valencia, Spain; pcumplido@aimplas.es

* Correspondence: amparo.chafer@uv.es (A.C.); jose.badia@uv.es (J.D.B.)

S1. Proposed mechanism of the degradation of PET

According to the literature [1], the proposed mechanism of the degradation of PET in a [bmim]-based ionic liquid is shown in **Figure S1**.

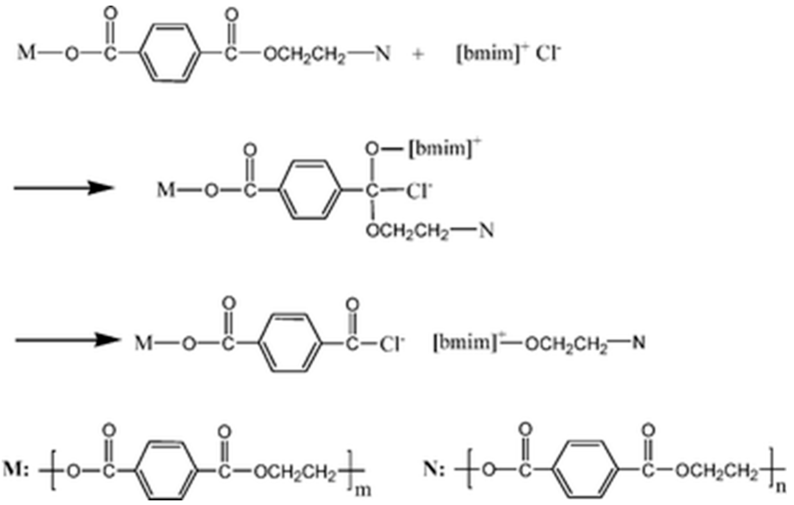

Figure S1. Possible mechanism of the degradation of PET in [bmim]Cl. Reproduced with permission from Hui Wang *et al.*, Green Chemistry; published by Royal Society of Chemistry, 2009 [1].

S2. List of experiments carried out under a factorial DoE statistical design of experiments

Table S1 gathers the list of experiments carried out under a 2^3 factorial DoE statistical design of experiments. This strategy was applied both for [Emim][OAc] and [Bmim][OAc] ionic liquids.

Table S1. DOE experimental planning. Note that the table has been ordered for the sake of clarity, but experiments were conducted following an aleatory sequence.

Run	Block	T	P/IL	P/S
**1**	1	170	4:1	1:2.5
**2**	1	170	4:1	1:5
**3**	1	170	4:1	1:7.5
**4**	1	170	2:1	1:2.5
**5**	1	170	2:1	1:5
**6**	1	170	2:1	1:7.5
**7**	1	170	1:1	1:2.5
**8**	1	170	1:1	1:5
**9**	1	170	1:1	1:7.5
**10**	1	180	4:1	1:2.5
**11**	1	180	4:1	1:5
**12**	1	180	4:1	1:7.5
**13**	1	180	2:1	1:2.5
**14**	1	180	2:1	1:5
**15**	1	180	2:1	1:7.5
**16**	1	180	1:1	1:2.5
**17**	1	180	1:1	1:5
**18**	1	180	1:1	1:7.5
**19**	1	190	4:1	1:2.5
**20**	1	190	4:1	1:5
**21**	1	190	4:1	1:7.5
**22**	1	190	2:1	1:2.5
**23**	1	190	2:1	1:5
**24**	1	190	2:1	1:7.5
**25**	1	190	1:1	1:2.5
**26**	1	190	1:1	1:5
**27**	1	190	1:1	1:7.5
**28**	2	170	4:1	1:2.5
**29**	2	170	4:1	1:5
**30**	2	170	4:1	1:7.5
**31**	2	170	2:1	1:2.5
**32**	2	170	2:1	1:5
**33**	2	170	2:1	1:7.5

Run	Block	T	P/IL	P/S
**34**	2	170	1:1	1:2.5
**35**	2	170	1:1	1:5
**36**	2	170	1:1	1:7.5
**37**	2	180	4:1	1:2.5
**38**	2	180	4:1	1:5
**39**	2	180	4:1	1:7.5
**40**	2	180	2:1	1:2.5
**41**	2	180	2:1	1:5
**42**	2	180	2:1	1:7.5
**43**	2	180	1:1	1:2.5
**44**	2	180	1:1	1:5
**45**	2	180	1:1	1:7.5
**46**	2	190	4:1	1:2.5
**47**	2	190	4:1	1:5
**48**	2	190	4:1	1:7.5
**49**	2	190	2:1	1:2.5
**50**	2	190	2:1	1:5
**51**	2	190	2:1	1:7.5
**52**	2	190	1:1	1:2.5
**53**	2	190	1:1	1:5
**54**	2	190	1:1	1:7.5

S3. Kinetic analysis based on the shrinking-core model

The reaction rate of the PET glycolysis catalysed by ionic liquids tested in this work was also modelled based on the shrinking-core theory which states that PET depolymerisation by glycolysis occurs in the solid-liquid interphase, i.e. a heterogeneous reaction, from surface to core with no shape changing of particles. This phenomenon has been usually reported [2]. Thus, the shrinking-core model is based on the **Equation S1** [3].

$$\left(\frac{1}{1-\frac{X_{PET}}{100}}\right)^{2/3} = k't \qquad \textbf{(S1)}$$

where k′ is the kinetic constant based on the shrinking-core model. The results of the kinetic analysis based on the shrinking-core model are shown in **Figure S2** and **Table S2**. This model was not able to adequately predict the reaction rate, especially at high temperatures since the results showed a different tendency. Thus, the evaluated catalytic glycolysis of PET in this work was mainly governed by a first-order kinetic, indicating that the reaction took place in a solution, i.e. a homogeneous system [3].

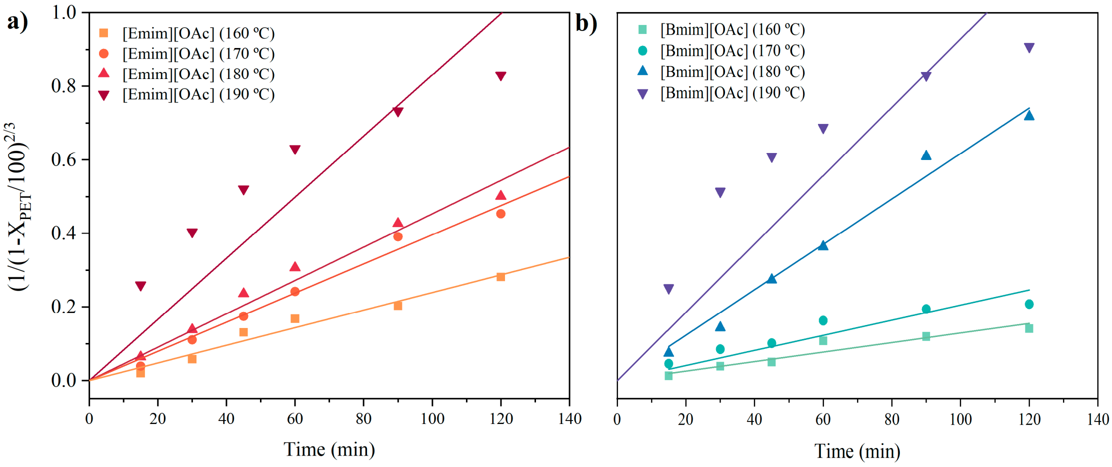

Figure S2. Effect of temperature on the conversion rate of PET using (**a**) [Bmim][OAc] and (**b**) [Emim][OAc] as catalysts at different temperatures. Tests conducted with 2, 5 and 2 g of PET, ethylene glycol and ionic liquid, respectively. Kinetic analysis based on the shrinking-core model.

Table S2. Kinetic coefficients (*k*) and lineal correlation coefficients (R^2) obtained from the kinetic analysis using the first-order model at different temperatures (T) for both ionic liquids [Bmim][OAc] and [Emim][OAc]. Kinetic analysis based on the shrinking-core model.

Ionic liquid	T (°C)	Equation	R^2
[Bmim][OAc]	160	$y = 1.29 \cdot 10^{-3} \cdot x$	0.9057
	170	$y = 2.05 \cdot 10^{-3} \cdot x$	0.8104
	180	$y = 6.17 \cdot 10^{-3} \cdot x$	0.9829
	190	$y = 9.29 \cdot 10^{-3} \cdot x$	0.4059
[Emim][OAc]	160	$y = 2.39 \cdot 10^{-3} \cdot x$	0.9614
	170	$y = 3.96 \cdot 10^{-3} \cdot x$	0.9832
	180	$y = 4.53 \cdot 10^{-3} \cdot x$	0.9682
	190	$y = 8.31 \cdot 10^{-3} \cdot x$	0.5122

References

1. Wang, H.; Li, Z.; Liu, Y.; Zhang, X.; Zhang, S. Degradation of Poly(Ethylene Terephthalate) Using Ionic Liquids. *Green Chemistry* **2009**, *11*, 1568–1575, doi:10.1039/B906831G.

2. Liu, Y.; Yao, X.; Yao, H.; Zhou, Q.; Xin, J.; Lu, X.; Zhang, S. Degradation of Poly(Ethylene Terephthalate) Catalyzed by Metal-Free Choline-Based Ionic Liquids. *Green Chemistry* **2020**, *22*, 3122–3131, doi:10.1039/D0GC00327A.

3. Li, J.; Zhang, S.; Li, H.; Ouyang, X.; Huang, L.; Ni, Y.; Chen, L. Cellulase Pretreatment for Enhancing Cold Caustic Extraction-Based Separation of Hemicelluloses and Cellulose from Cellulosic Fibers. *Bioresour Technol* **2018**, *251*, 1–6, doi:10.1016/J.BIORTECH.2017.12.026.
